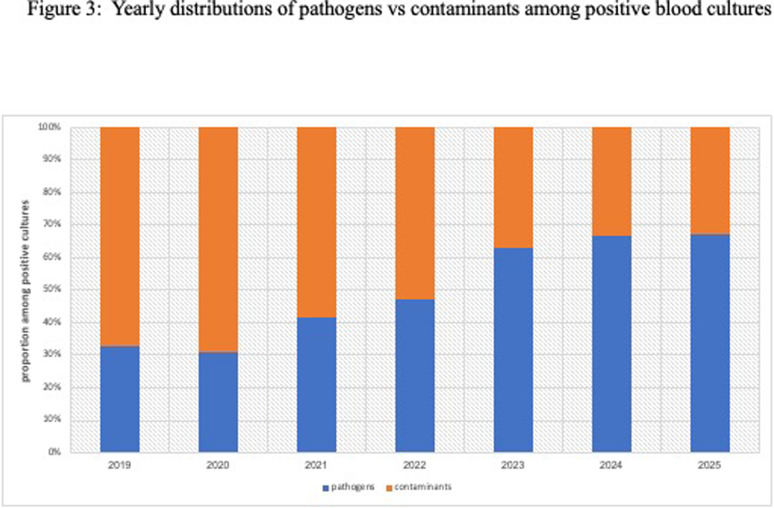# 192 Emerging opportunist or clinical harbinger? A review of Candida kefyr cases in Cancer Patients

**DOI:** 10.1017/ash.2026.10582

**Published:** 2026-06-23

**Authors:** Debby Ben David, Yael Cohen, Malka Yokobov, Maya Heled Akiva, Orana Schwartz, Yakov Nassimov, Andrey Chopen

**Affiliations:** 1 Wolfson Medical Center; 2 wolfson medical center; 3 Wolfson medical center

## Abstract

**Background** Blood cultures obtained in the emergency department (ED) are critical for diagnosing bloodstream infections but are frequently compromised by suboptimal sampling practices, contamination, and delays in transport to the microbiology laboratory. These limitations reduce diagnostic yield and may contribute to inappropriate antibiotic use. We evaluated the impact of a sustained, multicomponent quality-improvement intervention on blood culture quality, diagnostic yield, timeliness of transport to the microbiology laboratory, and time to culture growth. Methods We conducted a retrospective analysis of all blood cultures obtained in the ED of an acute-care hospital between January 2019 and December 2025. The ED treats approximately 85,000 patients annually. A multicomponent intervention was initiated in January 2021 and included repeated staff education, quarterly performance feedback, and workflow optimization. Blood culture transport was transitioned from manual delivery to a pneumatic tube system, and diversion tubes were introduced in January 2023 to decrease contamination. Outcomes included annual uptake of two-set sampling, pathogen detection, and contamination rates. Time from collection to incubation and growth was evaluated before and after the intervention. Trends across years were assessed using a test for linear trend. Results A total of 10,729 blood cultures were analyzed. Adoption of two-set blood culture sampling increased over time, from <3.2% (33/901) in 2019 to 45.9% (904/1,971) in 2025 (p<0.001; Figure 1). Overall, the proportion of blood cultures yielding a pathogen increased during the study period, rising from 6.8% (61/901) in 2019 to 12.2% (240/1,971) in 2025 (p for trend <0.001). Pathogen detection was significantly higher when two blood culture sets were obtained compared with a single set (12.9% [455/3,532] vs 8.8% [634/7,197]; p<0.001). In parallel, contamination rates declined markedly, from 13.9% (130/935) in 2019 to 6.0% (173/2,894) in 2025 (Figure 2). Among positive blood cultures, the distribution shifted over time, with contaminants predominating early and true pathogens predominating during the intervention period. (Figure 3) Timeliness of blood culture handling improved concurrently. Median time from collection to laboratory arrival decreased from 9.4 hours (IQR 2.8–14.9) to 2.3 hours (IQR 1.3–4.0; p<0.001). Concurrently, median time from collection to culture growth decreased from 27.7 hours (IQR 20.4–38.5) to 20.0 hours (IQR 14.6–29.3; p<0.001). Conclusions A sustained, multicomponent quality-improvement intervention in the ED was associated with improved blood culture diagnostic yield, reduced contamination, shorter laboratory processing times, and earlier growth detection. These findings highlight the value of coordinated process optimization to advance diagnostic stewardship and patient safety in high-acuity settings.

**Figure:**
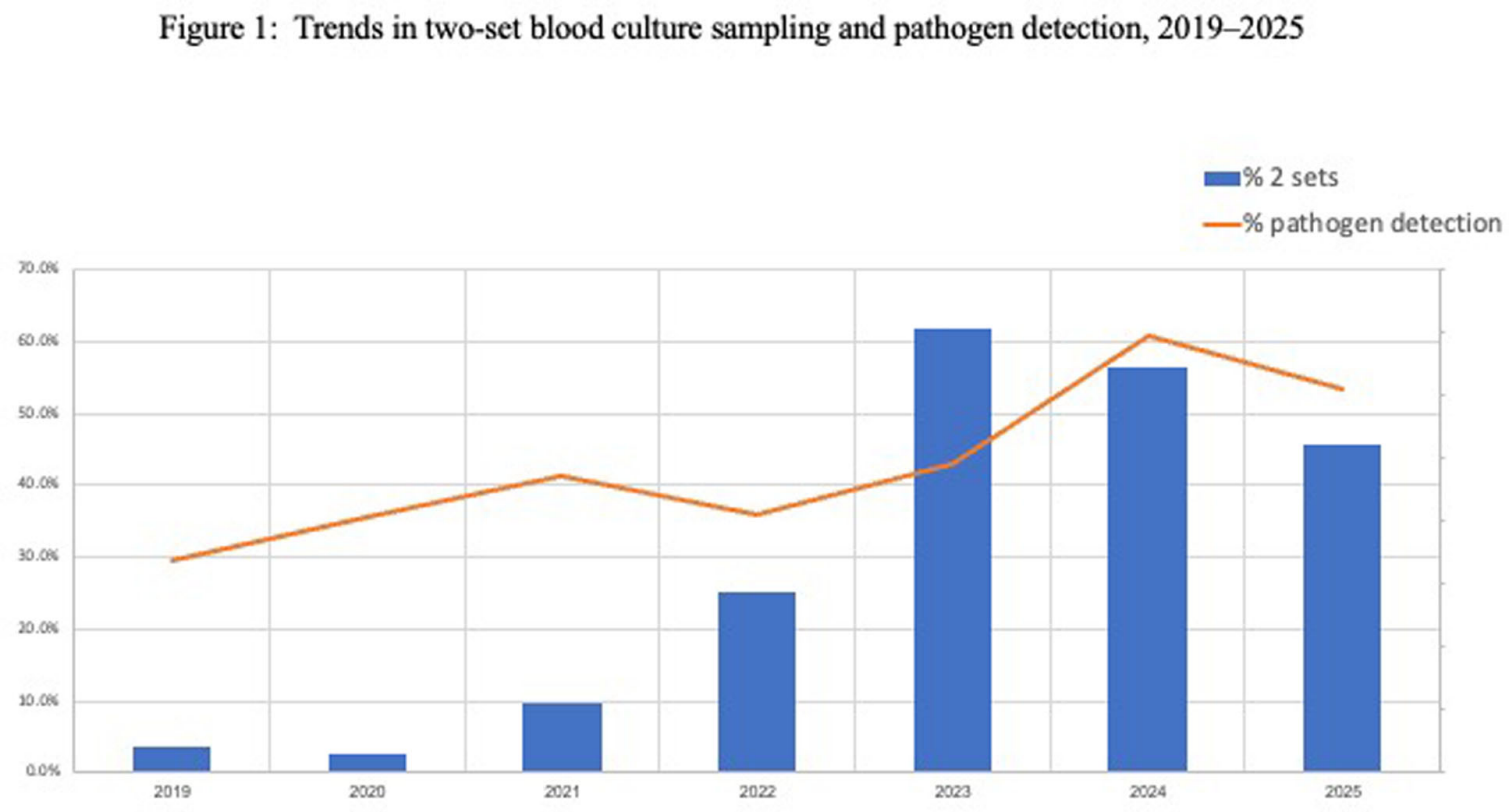

**Figure:**
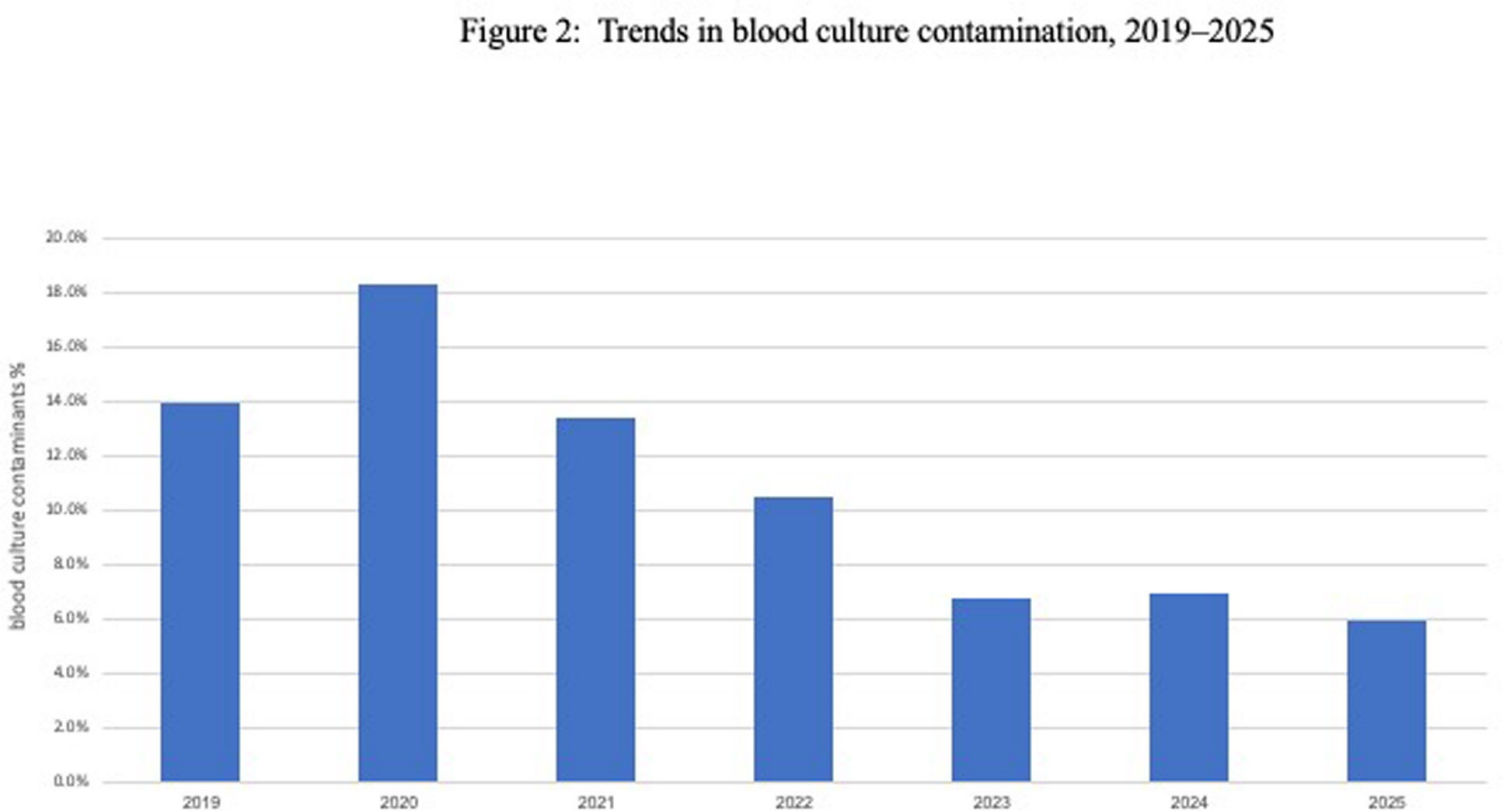

**Figure:**